# Prevalence of physical health comorbidities and long-term functional outcomes among community-reintegrated veterans following lower limb amputation in Sri Lanka

**DOI:** 10.1136/military-2023-002578

**Published:** 2023-11-21

**Authors:** Ashan Wijekoon, D Gamage Dona, S Jayawardana

**Affiliations:** 1 Department of Allied Health Sciences, Faculty of Medicine, University of Colombo, Colombo, Sri Lanka; 2 NICS-MORU, Colombo, Sri Lanka

**Keywords:** health & safety, limb reconstruction, musculoskeletal disorders, rehabilitation medicine

## Abstract

**Introduction:**

Lower limb amputation (LLA) poses significant health challenges, including physical health comorbidities (PHCs) and functional limitations. Military veterans, who typically undergo traumatic LLA at a young age, endure these challenges for an extended period. Understanding the extent of these challenges is vital to designing tailored and feasible postamputation care for them. In this study, we evaluated the prevalence of PHCs and long-term functional outcomes among community-reintegrated veterans following LLA in Sri Lanka.

**Methods:**

A comparative cross-sectional study was conducted in five districts in Sri Lanka. Prevalence of PHCs and functional outcomes were compared between community-reintegrated veterans with war-related traumatic LLA and a matched able-bodied cohort. Data on PHCs were collected from participants’ medical records and through a self-administered questionnaire. Timed-Up-and-Go (TUG) and 2 min walk test (2MWT) were used to compare functional outcomes between the groups. Veterans’ functional level was identified using the K-level classification.

**Results:**

Veterans were active prosthetic users who had undergone LLA >10 years ago. Sixty-six (77.6%) veterans reported experiencing phantom limb pain. A significantly higher prevalence of diabetes mellitus (34.2%), hypertension (22.4%), knee osteoarthritis (18.8%), knee pain (20%) and back pain (69.4%) was observed among veterans compared with the able-bodied group (p<0.05). Veterans demonstrated significantly lower levels of functional mobility (2MWT: mean (SD): 113.6 (14.8); increased risk of falling (TUG): mean (SD): 10.6 (1.8)) compared with able-bodied individuals (150.8 (11.9) and 7.2 (0.9), respectively, p<0.001). The majority of the veterans belonged to the K3 functional level (71.8%).

**Conclusions:**

The higher prevalence of PHCs and impaired functional outcomes underscores the multifaceted health challenges faced by veterans with LLA living in low-resource community settings with limited access to rehabilitation. These findings provide insights into the unique rehabilitation needs of individuals with similar backgrounds, informing the design and implementation of tailored rehabilitation interventions.

**WHAT IS ALREADY KNOWN ON THIS TOPIC**

- Physical health comorbidities (PHCs) and functional limitations are common health challenges experienced by individuals following lower limb amputation (LLA).
- This study addresses the scarcity of evidence on the prevalence and extent of these health challenges in specific LLA subgroups by focusing on military veterans living in low-resource community settings with limited access to rehabilitation who experienced war-related traumatic LLA over a decade ago.

**WHAT THIS STUDY ADDS**

- Our findings reveal a higher prevalence of PHCs in this group, including phantom limb pain (77.6%), back pain (69.4%), diabetes mellitus (34.2%), hypertension (22.4%), knee pain (20%) and knee osteoarthritis (18.8%), when compared with a matched able-bodied group.
- Most veterans belonged to the K3 functional level and demonstrated poor outcomes in functional mobility, with an increased risk of falling.

**HOW THIS STUDY MIGHT AFFECT RESEARCH, PRACTICE OR POLICY**

- The study’s findings highlight the unmet rehabilitation needs in this subgroup of LLA population, emphasising the importance of ensuring lifelong accessible rehabilitation services for individuals with LLA in similar contexts.

## Introduction

 Lower limb amputation (LLA) is a surgical procedure indicated for various conditions, including trauma, peripheral vascular disease and malignancy.^1^ While the primary objective of surgery is to address the underlying pathology and enhance mobility, individuals having undergone LLA often contend with substantial physical health challenges.^2^ These challenges include (1) the presence of chronic physical health comorbidities secondary to amputation, such as phantom limb pain (PLP), back pain, knee osteoarthritis, knee pain and diabetes mellitus; and (2) impaired functional outcomes, which significantly affect the overall quality of life of the affected individuals.^3^ Understanding these comorbidities and the amputation’s impact on long-term functional outcomes among a specific group of individuals following LLA is vital to designing tailored postamputation care and improving their overall well-being.

Individuals who undergo LLA due to trauma, such as combat injuries or other military-related incidents, commonly endure these challenges for an extended period of time, as their amputations typically occur during their young age.^4^ Sri Lanka, a nation with a history of civil war, is home to a substantial number of veterans who have undergone LLA. The estimated population of young veterans living with disabilities in Sri Lanka exceeds 20 000, with LLA, either independently or in conjunction with additional injuries, prevailing as the most common disability among them.^5^

Continuous engagement in physical rehabilitation has proven to be effective in improving physical health outcomes following LLA.^6^ However, rehabilitation opportunities for Sri Lankan veterans were limited to postinjury referrals for hospital-based rehabilitation, typically spanning a duration of 4–6 weeks, until their eventual reintegration into the community.^5^ Previous investigations have revealed a significant diminution of their quality of life, both in terms of physical and mental outcomes, compared with a demographically similar population without amputation.^7^ Furthermore, this decline in quality of life exacerbates over time, highlighting the unmet rehabilitation requisites for this cohort and underscoring the pressing need to develop accessible rehabilitation interventions.^8^ In order to enhance the feasibility and acceptability, it is important to tailor such interventions to the current state of their physical health outcomes, which remains unknown.

Hence, the objective of this study was to comprehensively determine the prevalence of physical health comorbidities and the long-term functional outcomes among community-reintegrated veterans following LLA in Sri Lanka. By examining these factors, we aim to contribute to the development of effective and tailored rehabilitation interventions for similar population groups, aimed at improving their overall well-being.

## Methods

### Study design

As part of a large-scale, mixed-method cross-sectional study, we investigated the prevalence of physical health comorbidities and functional outcomes among veterans with LLA compared with an age, sex and geographical location matched able-bodied cohort. The study was conducted in five districts of Sri Lanka, identified based on a priori knowledge of the location of veterans’ community settlements.

### Participants

We identified potential veterans from the ‘Disabled Category Registry’ obtained from the Directorate of Rehabilitation, Ministry of Defence, Sri Lanka. We selected participants from each district proportional to the number of potential veterans living in each district using a stratified random sampling procedure. For the veterans (study) group, we selected participants who had undergone unilateral LLA due to a battle injury, living in the community and using a prosthetic limb for ambulation. For the comparison group, we included age-matched and sex-matched (to veterans with LLA) able-bodied (non-amputee) individuals. These participants were identified from the same village or neighbouring village to the corresponding participant from the veterans’ group using the voter registration list.

### Prevalence of physical health comorbidities

To determine the prevalence of physical health comorbidities, the prevalence of PLP, diabetes mellitus, hypertension, knee osteoarthritis, knee pain and back pain was evaluated. Information on the prevalence of diabetes mellitus, hypertension and knee osteoarthritis was collected from participants’ medical records after obtaining their written informed consent. Participants were classified as having knee pain if they suffered knee pain in the intact leg at least 15 days in the previous month, either continuously or intermittently.^9^ For back pain, participants were asked if they experienced persistent, bothersome back pain at least once a week for the past 3 months.^10^ Questions regarding PLP were asked only from the veterans group. PLP was defined as painful sensation in the amputated part of the leg. The presence of PLP was recorded if participants reported experiencing it at least once in the month preceding data collection.^11^

### Functional outcomes

Functional outcomes were evaluated using the 2 min walk test (2MWT) and the Timed-Up-and-Go (TUG) test. Each participant was assigned to a functional level (K-level) based on the results of the TUG test. Detailed descriptions and demonstrations of all physical performance tests were provided by AW, and written consent was obtained from each participant before the tests were administered. All the tests were performed by the participants wearing the prosthesis. AW guided the participant throughout the test procedure and assisted in cases of emergencies (tendency to fall).

#### 2 min walk test

The 2MWT is a functional outcome measure widely used to evaluate functional mobility in individuals with LLA. It has been shown to correlate with measures of physical function and prosthetic use in this population, and significant differences were found in the performance of 2MWT between causes and levels of amputation.^12^ To perform the test, participants were instructed to walk along a rectangular pathway (15 m length and 0.58 m width) for 2 min, aiming to cover as much distance as possible without running or hopping, following the published guidelines.^12 13^ Distance covered within 2 min (2MWD) was recorded.

#### Timed-Up-and-Go test

The TUG test was used to evaluate the risk of falling among the participants.^14^ It has been validated to measure the risk of falling among individuals with unilateral LLA.^15^ The test was started with the participant seated on a chair (with seat height approximately 46 cm). The participant was instructed to stand up and start walking with the command ‘Go’ and walk 3 m and return back to the chair in a safe and comfortable speed on a carpet with 4 m length and 1 m width. Time taken (from the initial seated position to the first contact of the participant’s buttock to the seat) for the task was recorded using a stopwatch.

### Functional level (K-levels)

Functional level was determined using the K-level classification. The K-level score or the Medicare Functional Classification Level (MFCL) is an index for classifying the functional mobility and rehabilitation potential of people with LLA.^16 17^ The MFCL tool contains five categories (K-levels) sequenced in the order of increasing functional mobility (K0–K4), and each category defines the related functional level (table 1).

**Table 1:** K-level definitions and relevant Timed-Up-and-Go test values

K-level	Definition	Related Timed-Up-and-Go test score (s)
K0	Does not have the ability or potential to ambulate or transfer safely with or without assistance, and a prosthesis does not enhance quality of life or mobility.	<9.45*
K1	Has the ability or potential to use a prosthesis for transfers or ambulation in level surfaces at a fixed cadence. Typical of the limited and unlimited household ambulator.	
K2	Has the ability or potential for ambulation with the ability to transverse low-level environmental barriers such as curbs, stairs or uneven surfaces. Typical of the limited community ambulator.	
K3	Has the ability or potential for ambulation with variable cadence. Typical of the community ambulator who has the ability to transverse most environmental barriers and may have vocational, therapeutic or exercise activity that demands prosthetic use beyond simple locomotion.	9.45–12.82
K4	Has the ability or potential for prosthetic ambulation that exceeds basic ambulation skills, exhibiting high impact, stress or energy levels. Typical of the prosthetic demands of the child, active adult or athlete.	>12.82

* Participants with K0, K1, or K2 levels were not included in the referenced study. Therefore, scores below 9.45 seconds s were considered as having functional level below K3.

However, due to its subjectivity, it is difficult to assign patients into each level depending only on the level definitions. Therefore, we used the results of the TUG test to assign K-levels to each participant based on a study conducted by Sions *et al*^17^ (table 1). Their findings suggest that the TUG test score distinguishes between different functional levels and can be used in the objective assignment of K-levels in the unilateral LLA population.

### Data analysis

Statistical analyses were carried out by DGD, a certified statistician with no involvement in participant allocation or data collection, using STATA/IC for Mac V.16.1. Data summaries included mean values with SD for participant characteristics and functional outcomes, and counts with percentages for prevalence of comorbidities and functional levels. Shapiro-Wilk test was employed to assess the normality of data distribution. Group comparisons for continuous variables were conducted using independent sample t-test, while nominal variables were assessed with χ^2^ test, adopting a significance level of 0.05.

## Results

### Characteristics of participants

In total, 170 individuals (85 in each group) participated in the study. All the veterans were men with unilateral transfemoral or transtibial LLA, with age ranging from 30 to 55 years (mean (SD): 46.3 (6.0)) (table 2). For all the veterans, the cause of amputation was a battle injury that occurred more than 10 years ago (mean (SD): 21.7 (5.9)). They were all active prosthetic users living in the community with their families. All had completed initial postsurgical prosthetic training in the form of institutional care. There was no routine follow-up by rehabilitation providers, and all participants were currently not engaged in formal physical rehabilitation. There were no significant differences in terms of age and body mass index between the veterans with LLA and the able-bodied comparison group (p>0.05) (table 2).

**Table 2:** Demographic and clinical characteristics of the participants

Characteristics	Veterans with LLA (n=85)	Able-bodied group (n=85)
Gender, male (%)	100	100
Age, mean (SD)	46.3 (6.0)	46.7 (6.0)
BMI, mean (SD)	26.2 (3.4)	25.0 (3.1)
Time since amputation, mean (SD)	21.7 (5.9)	N/A
Amputation type, unilateral (%)	100	N/A
Amputation level (%)		
Transfemoral	8.2	N/A
Transtibial	91.7	

BMI, body mass index; LLA, lower limb amputation; N/A, not applicable.

### Prevalence of physical health comorbidities

**Figure 1:**
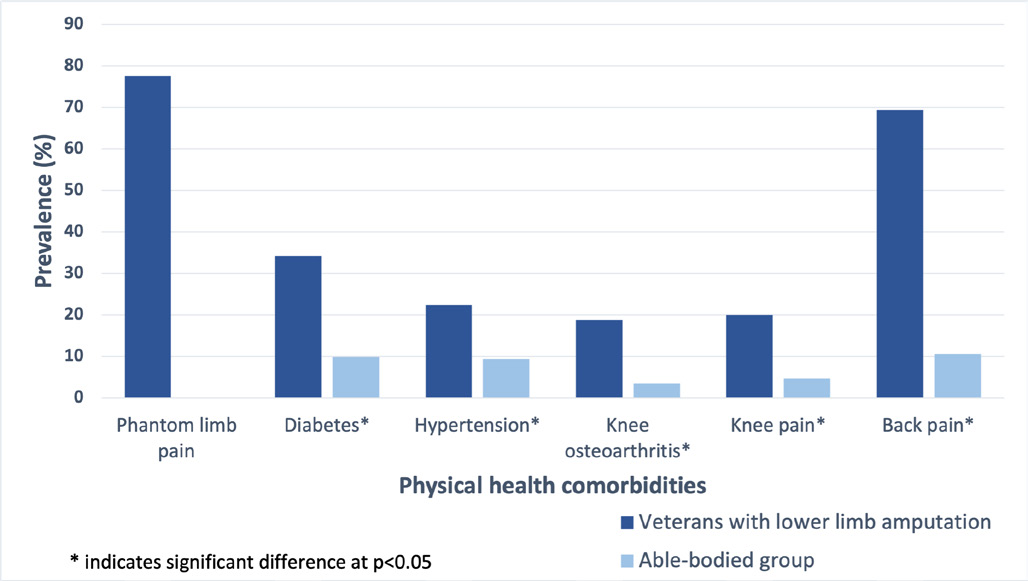
Comparison of the prevalence of physical health comorbidities between veterans with lower limb amputation and the able-bodied individuals.

Figure 1 illustrates the prevalence of different physical health comorbidities among the study participants. Veterans with LLA had a high prevalence of PLP (77.6%) experienced during the month preceding the data collection. A significantly higher prevalence of diabetes (34.2%) and back pain (69.4%) was observed among veterans with LLA compared with the able-bodied group (9.8% (p=0.003) and 10.6% (p<0.001), respectively). Other comorbidities also exhibited significantly higher prevalence among the veterans with LLA compared with the able-bodied individuals: hypertension (22.4% vs 9.4%, p=0.021), knee osteoarthritis (18.8% vs 3.5%, p=0.019) and knee pain (20% vs 4.7%, p<0.001).

### Functional outcomes

Table 3 presents the comparison of functional outcomes between the two groups. Veterans with LLA demonstrated significantly lower levels of functional mobility (lower 2MWD) and increased risk of falling (higher TUG test score) compared with the able-bodied group (p<0.001). When comparing between participants with different amputation levels, the transfemoral group had poorer outcomes in both functional outcomes, with a significant difference only in the TUG test score (p<0.001) (online supplemental table 1).

**Table 3:** Comparison of functional outcomes between veterans with lower limb amputation and the able-bodied individuals

Functional outcome	Outcome measure	Veterans with LLA, mean (SD)	Able-bodied group, mean (SD)
Risk of falling	Timed-Up-and-Go test (s)	10.6 (1.8)	7.2 (0.9)*
Functional mobility	2 min walk test (m)	113.6 (14.8)	150.8 (11.9)*

* Indicates significant difference in outcomes between the two groups at p<0.05.

LLA, lower limb amputation.

### Functional level

**Figure 2:**
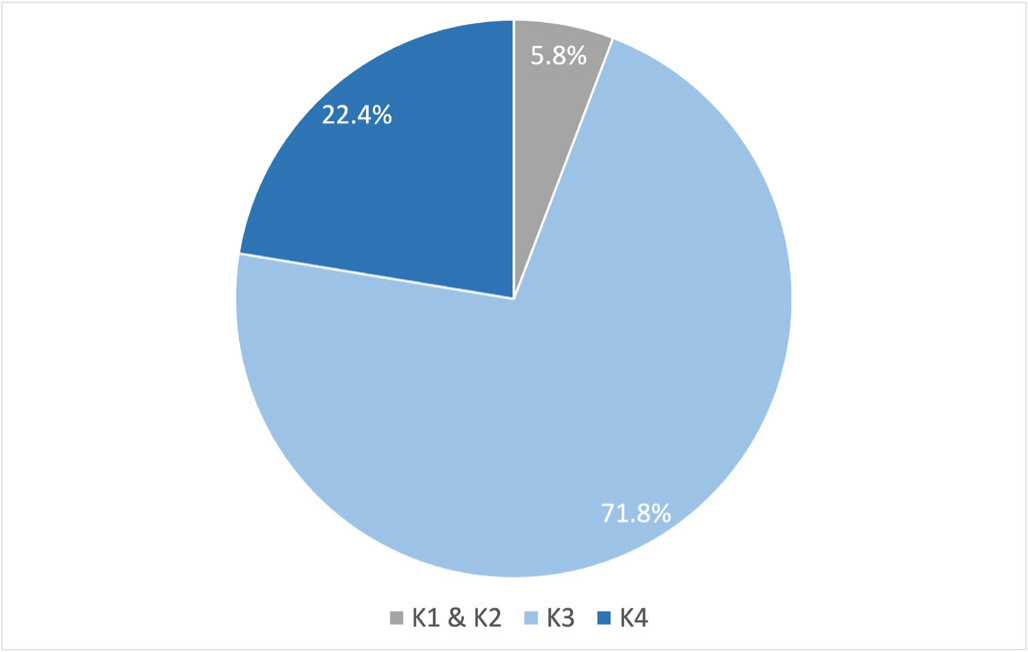
Distribution of veterans with lower limb amputation across different functional levels.

The majority of the veterans with LLA fell under functional levels K3 and K4, with the highest number in the K3 level (71.8%) (figure 2). Five (5.8%) veterans had functionality below K3 level, and among them four had transfemoral amputations (online supplemental table 1). According to the description of K-levels, these five veterans would fall into either the K2 or K1 functional level. This is because the study sample specifically included active prosthetic users, and K0 level represents the absence of ambulatory ability, with or without assistance (table 1). The distribution of these functional levels varied significantly between different amputation levels (p<0.001), and it is worth noting that all veterans with LLA who belonged to the highest functional level (K4 level) had transtibial amputations (online supplemental table 1).

## Discussion

The findings of this study highlight a higher prevalence of physical health comorbidities and lower functional outcomes among community-reintegrated veterans with a history of LLA spanning decades, when compared with their able-bodied counterparts.

In this study, a high prevalence of physical health comorbidities was observed among veterans with LLA. Specifically, 77% of participants reported experiencing PLP within the last month, despite the fact that PLP typically improves over time following amputation. Comparing our findings with a recent meta-analysis,^18^ the prevalence of PLP in our study population appears to be relatively higher. The meta-analysis reported a PLP prevalence ranging from 27% to 85.6%, with a pooled estimated prevalence of 64%. Additionally, the PLP prevalence found in our study was significantly higher than a similar military population with LLA (49%) who were receiving continuous outpatient rehabilitation care.^11^ This suggests that targeted interventions aimed at reducing PLP could be beneficial to the participants of the present study. Since PLP is influenced by psychological factors like stress and depression,^19^ this may also suggest the poor psychological well-being among these individuals.

Nearly 70% of the study participants had persistent back pain. This is consistent with previous studies reporting higher than 60% of back pain prevalence among individuals with traumatic LLA.^20 21^ Back pain even at a moderate level has a significant negative impact on functional ability and quality of life among individuals with LLA.^22^ The impact of back pain on functional ability is particularly important to consider. Back pain can limit mobility, affect posture and balance, and interfere with daily activities and functional independence. Additionally, the negative impact of back pain extends beyond physical limitations; it can affect psychological well-being and social participation and reduce overall satisfaction with life.^23^

Osteoarthritis and pain in the intact leg knee joint are common secondary comorbidities associated with unilateral LLA, contributing to limited functional mobility and reduced quality of life.^20^ In our study, participants exhibited a higher prevalence of these comorbidities compared with their able-bodied counterparts, highlighting their poor physical health outcomes. The altered gait mechanics observed among individuals with LLA, such as increased load on the intact leg, can lead to overuse and greater wear of joint structures and surrounding muscles.^24^ This adaptation aims to accommodate the limited power output and instability of the prosthetic leg during stance, ultimately contributing to the development of knee osteoarthritis and resultant knee pain. Therefore, future rehabilitation interventions targeting this population should focus on optimising the quality of the prosthetic leg and correcting gait mechanics.

The association between physical health comorbidities and functional ability is further supported by the significantly higher risk of falling (indicated by the TUG test) and lower functional mobility (indicated by the 2MWT) observed among veterans with LLA compared with the able-bodied group. Previous research by Gaunaurd *et al*^12^ demonstrated that individuals with a mean 2MWD of 130.6 had a high health risk based on waist to hip ratio indicating that the LLA population in our study may also have a heightened health risk. Furthermore, a TUG test score of >9.25 s has been identified as the cut-off value for the risk of experiencing two or more falls in individuals with unilateral LLA.^25^ In our study, the mean TUG test score among veterans with LLA was 10.6, suggesting an increased risk of falls within this population.

These findings suggest that the presence of comorbidities and the associated functional limitations contribute to reduced mobility and physical performance in this population. Additionally, the limited participation in physical activities resulting from these challenges^26^ may contribute to the higher prevalence of conditions such as diabetes mellitus and hypertension among veterans with LLA found in this study, as physical inactivity is a known risk factor for these conditions.^27 28^ Furthermore, the observed prevalence rates of diabetes mellitus and hypertension in our study were consistent with those reported in a previous study conducted in 1989 on a similar war-related traumatic LLA population.^29^ These consistent prevalence rates over several decades indicate that, despite advancements in healthcare services over these years, the studied population still exhibits higher rates of these conditions, paralleling similar populations from several decades ago.

However, it is important to note that, despite these challenges, the majority of the veterans in our study belonged to a functional level that has the ability to navigate various environmental conditions and actively engage in vocational, therapeutic or exercise activities that demand the use of a prosthetic limb beyond basic mobility. This highlights the resilience and adaptability of these individuals in participating in future meaningful physical rehabilitation programmes to maximise their functional capacity and overall well-being.

One limitation of this study is the exclusive inclusion of military veterans, limiting the external validity of the results to war-related traumatic LLA. Since we did not aim to purposively sample based on the amputation level, only a small number of participants with transfemoral amputation (7 out of 85) were included in the study, reducing the reliability of comparisons between transtibial and transfemoral amputations. However, the authors believe that the inclusion of participants adequately powered for outcome measures, using random stratified sampling, and the inclusion of a matched comparison group of civilians add significant value to the literature by describing the long-term health consequences of LLA in a specific disadvantaged group residing in a low-resource setting. Pertaining to the objective of the study, we focused solely on the prevalence of chronic pain. However, we recognise that incorporating data on the severity of these pains would have provided a more comprehensive understanding of participant experiences. It is also noteworthy that while the literature supports the allocation of participants to different functional levels based on performance-based tests, the potential deviations from a comprehensive clinical judgement should be considered when interpreting data on functional level.

## Conclusions

The higher prevalence of physical health comorbidities and impaired functional outcomes, compared with a group of matched able-bodied individuals, underscores the multifaceted health challenges faced by veterans with LLA in Sri Lanka, exposing them to increased health risks. This emphasises the importance of urgent implementation of comprehensive management strategies that address their rehabilitation needs. Further research is warranted to investigate the underlying mechanisms and risk factors contributing to the development of these comorbidities in the context of LLA, with the aim of informing effective preventive strategies and interventions tailored to the needs of this population. Our study provides valuable insights into the current physical health status of veterans with LLA living in communities with limited access to rehabilitation services in a low-resource setting. These insights enhance the understanding of their specific rehabilitation needs and challenges, which in turn will inform the design and implementation of targeted rehabilitation interventions for this population.

## Supplementary material

10.1136/military-2023-002578 online supplemental file 1

## Data Availability

Data are available upon reasonable request.

## References

[1] Ephraim PL, Dillingham TR, Sector M (2003). Epidemiology of limb loss and congenital limb deficiency: a review of the literature. Arch Phys Med Rehabil.

[2] Fortington LV, Rommers GM, Geertzen JHB (2012). Mobility in elderly people with a lower limb amputation: a systematic review. J Am Med Dir Assoc.

[3] Asano M, Rushton P, Miller WC (2008). Predictors of quality of life among individuals who have a lower limb amputation. Prosthet Orthot Int.

[4] Esfandiari E, Yavari A, Karimi A (2018). Long-term symptoms and function after war-related lower limb amputation: a national cross-sectional study. Acta Orthop Traumatol Turc.

[5] Kumara AA (2015). Rehabilitation management system for the directorate of rehabilitation of the Sri Lanka Army.

[6] Wijekoon A, Jayawardana S, Milton-Cole R (2023). Effectiveness and equity in community-based rehabilitation on pain, physical function, and quality of life following unilateral lower limb amputation: a systematic review. Arch Phys Med Rehabil.

[7] Gunawardena NS, Seneviratne R de A, Athauda T (2006). Functional outcomes of unilateral lower limb Amputee soldiers in two districts of Sri Lanka. Mil Med.

[8] Gowinnage SS, Arambepola C (2020). Quality of life and its determinants among community re-integrated soldiers with permanent disabilities following traumatic limb injuries. Qual Life Res.

[9] Norvell DC, Czerniecki JM, Reiber GE (2005). The prevalence of knee pain and symptomatic knee osteoarthritis among veteran traumatic Amputees and Nonamputees. Arch Phys Med Rehabil.

[10] Ehde DM, Smith DG, Czerniecki JM (2001). Back pain as a secondary disability in persons with lower limb amputations. Arch Phys Med Rehabil.

[11] Aldington D, Small C, Edwards D (2014). A survey of post-amputation pains in serving military personnel. J R Army Med Corps.

[12] Gaunaurd I, Kristal A, Horn A (2020). The utility of the 2-minute walk test as a measure of mobility in people with lower limb amputation. Arch Phys Med Rehabil.

[13] Bohannon RW, Wang YC, Gershon RC (2015). Two-minute walk test performance by adults 18 to 85 years: normative values, reliability, and responsiveness. Arch Phys Med Rehabil.

[14] Kear BM, Guck TP, McGaha AL (2017). Timed up and go (TUG) test. J Prim Care Community Health.

[15] Tanneke S (2011). TUG test reliability and validity in LLA. Acta Fisiátrica.

[16] Borrenpohl D, Kaluf B, Major MJ (2016). Survey of U.S. practitioners on the validity of the medicare functional classification level system and utility of clinical outcome measures for aiding K-level assignment. Arch Phys Med Rehabil.

[17] Sions JM, Beisheim EH, Manal TJ (2018). Differences in physical performance measures among patients with unilateral lower-limb amputations classified as functional level K3 versus K4. Arch Phys Med Rehabil.

[18] Limakatso K, Bedwell GJ, Madden VJ (2020). The prevalence and risk factors for phantom limb pain in people with amputations: a systematic review and meta-analysis. PLoS One.

[19] Sherman RA, Sherman CJ, Bruno GM (1987). Psychological factors influencing chronic phantom limb pain: an analysis of the literature. Pain.

[20] Robbins CB, Vreeman DJ, Sothmann MS (2009). A review of the long-term health outcomes associated with war-related amputation. Mil Med.

[21] Ephraim PL, Wegener ST, MacKenzie EJ (2005). Phantom pain, residual limb pain, and back pain in Amputees: results of a national survey. Arch Phys Med Rehabil.

[22] Hammarlund CS, Carlström M, Melchior R (2011). Prevalence of back pain, its effect on functional ability and health-related quality of life in lower limb Amputees secondary to trauma or tumour: a comparison across three levels of amputation. Prosthet Orthot Int.

[23] Sivapuratharasu B, Bull AMJ, McGregor AH (2019). Understanding low back pain in traumatic lower limb Amputees: a systematic review. *Arch Rehabil Res Clin Transl*.

[24] Zhang T, Bai X, Liu F (2019). Effect of prosthetic alignment on gait and Biomechanical loading in individuals with transfemoral amputation: a preliminary study. Gait Posture.

[25] Sawers A, Hafner BJ (2020). Using clinical balance tests to assess fall risk among established unilateral lower limb prosthesis users: cutoff scores and associated validity indices. PM R.

[26] Pepin M-E, Devour A, Coolsaet R (2019). Correlation between functional ability and physical activity in individuals with transtibial amputations: a cross-sectional study. Cardiopulm Phys Ther J.

[27] Naschitz JE, Lenger R (2008). Why traumatic leg Amputees are at increased risk for cardiovascular diseases. QJM.

[28] Powell KE, Thompson PD, Caspersen CJ (1987). Physical activity and the incidence of coronary heart disease. Annu Rev Public Health.

[29] Yekutiel M, Brooks ME, Ohry A (1989). The prevalence of hypertension, ischaemic heart disease and diabetes in traumatic spinal cord injured patients and Amputees. Paraplegia.

