# Supplementary material for: Prevalence of physical health comorbidities and long-term functional outcomes among community-reintegrated veterans following lower limb amputation in Sri Lanka
Source: BMJ Mil Health. 2023 Nov 21;171(2):e002578. doi: 10.1136/military-2023-002578 (PMC12015008; doi:10.1136/military-2023-002578)
Supplement: online supplemental file 1 [file military-171-2-s001.pdf]

Supplementary Table 1: Comparison of physical health comorbidities, functional outcomes, and functional levels between different amputation levels.

**Physical health comorbidities, functional outcomes, and functional levels**	**Transfemoral (n=7)**	**Transtibial (n=78)**
Physical health comorbidities (%)		
Phantom limb pain	71.4	78.2
Diabetes	42.8	33.3
Hypertension	14.3	23.1
Knee osteoarthritis	57.1	15.4
Knee pain	42.8	17.9
Back pain	71.4	69.2
Functional outcomes (Mean (SD))		
Functional mobility (2MWT)	94.3 (6.8)	115.4 (14.1)
Risk of falling (TUG test)*	14.3 (2.9)	10.3 (1.3)
Functional levels (%)*		
K1 and K2	42.8	2.3
K3	57.1	73.1
K4	0	24.6

2MWT, 2-minute walk test; TUG, timed-up-and-go.

* indicates significant difference in outcomes between transfemoral and transtibial at $p<0.05$ level
